# The Significance of Inflammatory Markers in Pediatric Patients with Acute Gastroenteritis Presenting to the Emergency Department

**DOI:** 10.3390/children12050617

**Published:** 2025-05-09

**Authors:** Hazem Alhazmi, Abeer Alzahrani, Saud Alshaikh, Lein Azzhary, Fatimah Alhaddad, Zeyad Alshamrani, Raghad Alwagdani

**Affiliations:** 1 Pediatric Emergency Department, NGHA, Jeddah 11426, Saudi Arabia; 2 King Abdullah’s Specialized Children Hospital, Jeddah 11426, Saudi Arabia; 3 Prince Sultan Military Medical City, Riyadh 12233, Saudi Arabia

**Keywords:** acute gastroenteritis, inflammatory markers, white blood cells counts, pediatric emergency medicine

## Abstract

**Background:** Considerable mortality and morbidity rates linked to AGE are well documented in the literature. Many inflammatory markers have been studied in the context of research on AGE as tools to predict the clinical course of the disease and determine the need for the use of antimicrobials. This study focuses on CRP, PCT, and WBC counts as inflammatory markers of AGE. **Methods:** A retrospective chart review study was conducted at King Abdullah Specialized Children’s Hospital, Jeddah. Using a non-probability consecutive sampling technique, all patients under the age of 14 diagnosed with gastroenteritis over four years (2020–2024) were included. **Results:** The sample population consisted of 84 individuals. Pathogen prevalence was identified in only 15%. *Salmonella* was the most frequently identified bacterial pathogen. While the WBC count and ESR were reassuring in most cases, the CRP and PCT measurements were almost always elevated. Compared to the stronger association observed with WBC counts, the correlation between PCT levels and ED visits were less significant. Higher CRP levels were associated with an increased use of antibiotics. **Conclusion:** The results of this study highlight that CRP is useful in identifying patients who are likely to have bacterial AGE and require antibiotics. Moreover, the WBC count is a helpful tool in predicting those likely to present to the ED again.

## 1. Introduction

Acute gastroenteritis (AGE) remains one of the most common reasons for pediatric emergency department (ED) visits worldwide and often leads to significant morbidity and healthcare resource utilization. The clinical presentation of AGE can vary widely, ranging from mild, self-limiting symptoms to severe dehydration and systemic inflammation. Inflammatory markers, such as C-reactive protein (CRP) and procalcitonin (PCT), have emerged as valuable tools in the clinical decision-making process, providing critical insights into the severity of inflammation and guiding treatment strategies for pediatric patients presenting with AGE.

Recent studies have described the utility of inflammatory biomarkers in assessing the severity of pediatric diarrhea. For instance, Park et al. (2019) demonstrated that elevated levels of fecal calprotectin and lactoferrin are associated with more severe presentation of diarrhea, suggesting that these markers can help identify patients who are likely to require further supportive measures like fluid resuscitation or anti-inflammatory therapies [1].

The European Society for Pediatric Gastroenterology, Hepatology, and Nutrition (ESPGHAN) published guidelines for the management of cases of AGE in 2014 [2]. Although the use of laboratory workups is not routinely recommended, these guidelines shine light on their role in differentiating between bacterial and non-bacterial infections. The publication states that the evidence suggests the usefulness of both CRP and PCT, with the latter being the more promising tool. However, the evidence is low-quality due to the lack of studies investigating CRP and PCT in the context of AGE, thus rendering the use of CRP and PCT a weak recommendation. The article also emphasizes that the use of antimicrobials in AGE is not routinely recommended, except under certain conditions. These conditions include the following: an age of <3 months, immune deficiency, immunosuppressive therapy, anatomical or functional asplenia, bacteremia, and inflammatory bowel disease. Furthermore, some organisms, including *Shigella*, *Enterotoxigenic E.coli*, and *dysenteric Campylobacter*, warrant antimicrobial treatment, as well.

To further refine risk stratification, Levine et al. (2022) developed the Pediatric Acute Gastroenteritis Risk Score, which incorporates clinical and laboratory parameters, including inflammatory markers, to predict the likelihood of moderate to severe AGE. This scoring system emphasizes the importance of integrating inflammatory markers into clinical assessments to optimize patient care and resource allocation [3]. However, the role of procalcitonin in AGE remains less clear. While procalcitonin has been studied in the context of inflammatory bowel disease, its association with disease severity in AGE is limited to small-scale case-control studies and thus requires further research [4].

The American Academy of Pediatrics (AAP) has also addressed the management of AGE in children. It has emphasized the importance of clinical assessment and the judicious use of laboratory testing. The AAP guidelines recommend against routine testing for viral or bacterial pathogens in uncomplicated cases but acknowledge the potential value of inflammatory markers such as CRP in severe or atypical presentations [5]. Additionally, a systematic review by Freedman et al. (2016) highlighted the predictive value of CRP in distinguishing bacterial from viral gastroenteritis, further supporting its role in guiding treatment decisions [6].

Despite these advancements, there is limited data on how CRP and procalcitonin correlate with viral versus bacterial etiologies of AGE. Furthermore, the patterns and trends of these markers have not been systematically evaluated in the context of pediatric AGE management and disposition decisions in the ED. Understanding these relationships could provide pediatricians with evidence-based guidance on the necessity and timing of incorporating CRP and procalcitonin into clinical practice.

## 2. Materials and Methods

**Figure 1:**
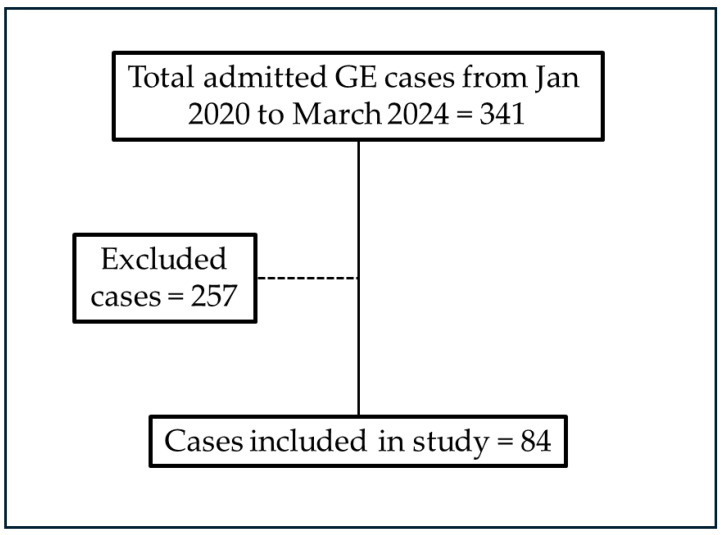
Patient enrollment flow graph.

A retrospective chart review study was conducted at King Abdullah Specialized Children’s Hospital, Jeddah (KASCH-JD). All patients diagnosed with gastroenteritis over four years (2020–2024) were identified, and their health records were obtained using the hospital’s health information system, BESTCare. As shown in Figure 1, using a non-probability consecutive sampling technique, we included all patients under the age of 14 years who were diagnosed with acute gastroenteritis. We excluded patients with clinical elements that may have impacted the management of their care or clinical decision-making. These included cases with presumed or confirmed surgical abdomen, such as appendicitis or intussusception; chronic diarrheas, such as congenital secretory diarrhea or malabsorption; syndromes associated with gastrointestinal disturbances or anatomical abnormalities, such as short bowel syndrome; and patients with immunodeficiency, endocrinopathy, or gastrointestinal diseases, such as hyperthyroidism, inflammatory bowel disease, and celiac disease.

The data collection sheet included age, gender, medical background, presenting symptoms and signs, initial investigations, extraintestinal infections, and the management offered. Gastroenteritis was defined by the treating team as a discharge diagnosis and further reaffirmed as such during data collection. The presenting symptoms included the following: frequent vomiting and diarrhea, the presence of fever, abdominal pain, and bloody diarrhea. The duration of symptoms and number of emergency department visits were also taken into account. Other presentation-related data included initial vitals and the severity of dehydration. The investigations that were included were of the white blood cell (WBC) count and WBC differentials, the neutrophils (Neuts) and lymphocytes (Lymphs); C-reactive protein (CRP); procalcitonin (PCT); and the erythrocyte sedimentation rate (ESR). Pathogen detection tests were also included if they had been performed. These are stool cultures and microbiological stool analyses by PCR. The presence of extraintestinal infections was identified by symptoms, and infections were furthermore confirmed by treating physicians as being secondary upper respiratory tract infections (URTIs), lower respiratory tract infections (LRTIs), urinary tract infections (UTIs), or sepsis. Management lines included an oral challenge with/without antiemetic, normal saline bolus, dextrose 10% water bolus, maintenance/deficit IV fluid, and antibiotics.

Data analysis was carried out using PRISM version 9. For descriptive statistics, frequency and percentage were computed for the categorical data. Numerical data were presented with the mean and standard deviation. The study considered a confidence interval (CI) of 95%. A two-sided *p*-value of equal to or less than 0.05 was considered significant. The ethics committee at King Abdullah International Medical Research Center granted ethical approval for the study. As data was collected from health records, informed consent was not applicable, and all patients’ medical record numbers were anonymized during data analysis.

## 3. Results

### 3.1. Demographic Characteristics

The sample population consisted of 84 individuals, as shown in Table 1. The distribution was relatively balanced. Most of the participants (60.71%) had a normal medical background. The remaining participants had illnesses that were not part of the exclusion criteria and did not alter the medical decisions or management that was offered. These conditions included prematurity, bronchial asthma, hemolytic anemias with no active hemolysis, epilepsy, developmental delay, down syndrome, and gene mutations with no related gastrointestinal manifestations, among other conditions.

### 3.2. Clinical Presentation and Summary of Inflammatory Markers

In terms of clinical presentation, the majority of patients (69%) had a single visit to the emergency department (ED) and a mean duration of symptoms of 2 days. The mean frequencies of diarrhea and vomiting were 4.64 and 3.70 episodes per day, respectively. Most of the patients who presented had fever, tachycardia, and a dehydration severity ranging from mild to moderate based on the clinical dehydration scale (CDS). Only a small number had bloody diarrhea, hypotension, or hypoglycemia at presentation. The inflammatory markers that were assessed included the following parameters: WBCs and WBC differentials, neutrophils and lymphocytes; CRP; PCT; and the ESR. Each marker is accompanied by its normal range for context. While the WBC count and WBC differentials, along with the ESR, were reassuring in most cases, other inflammatory markers, such as CRP and PCT, were almost always elevated. Further details regarding clinical presentations and inflammatory markers can be found in Table 2 and Table 3.

### 3.3. Etiology, Extraintestinal Infections, and Management Offered

**Figure 2:**
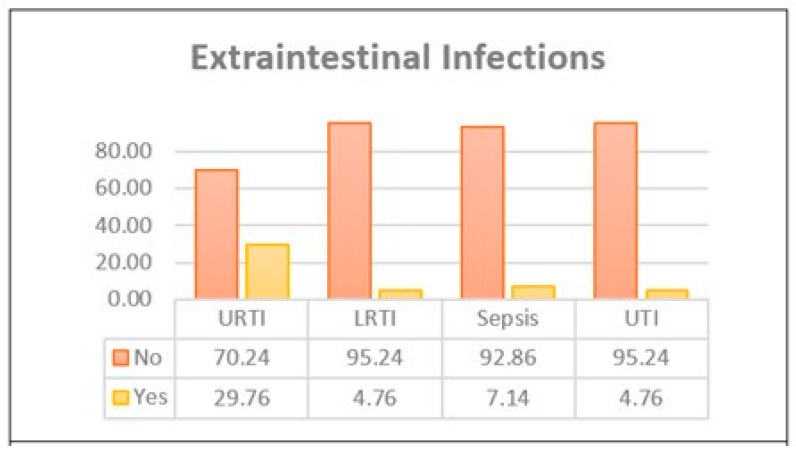
Percentage of cases with confirmed extraintestinal infections.

**Figure 3:**
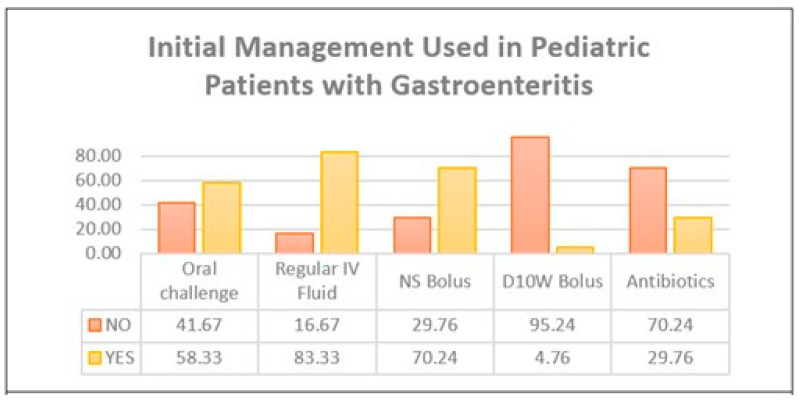
Percentages for types of initial management used in pediatric patients with gastroenteritis.

An underlying pathogen was identified in only 13 cases (15%). This is partly due to the lack of pathogen identification testing for all included samples. As described in Table 4, *Rotavirus* and *Norovirus* were identified in one case each, while *Salmonella* was the most frequently identified bacterial pathogen, occurring in six cases. Other pathogens that were identified were isolated bacteria, such as *Shigella*; mixed viruses; and bacterial organisms. Around one third of patients admitted with gastroenteritis had a concurrent URTI. In contrast, LRTIs, sepsis, and UTIs were rarely confirmed. These co-infections were confirmed clinically, radiologically, and microbiologically using cultures or PCR. While antibiotics were offered for one third of patients, the requirement of a dextrose 10% bolus was rare in our sample, as only a few patients had hypoglycemia. Figure 2 and Figure 3 further break down the percentages of extraintestinal infections and management lines offered.

### 3.4. Relationships of Inflammatory Markers with Duration of Symptoms and Numbers of ED Visits in Cases of Severe Dehydration

**Figure 4:**
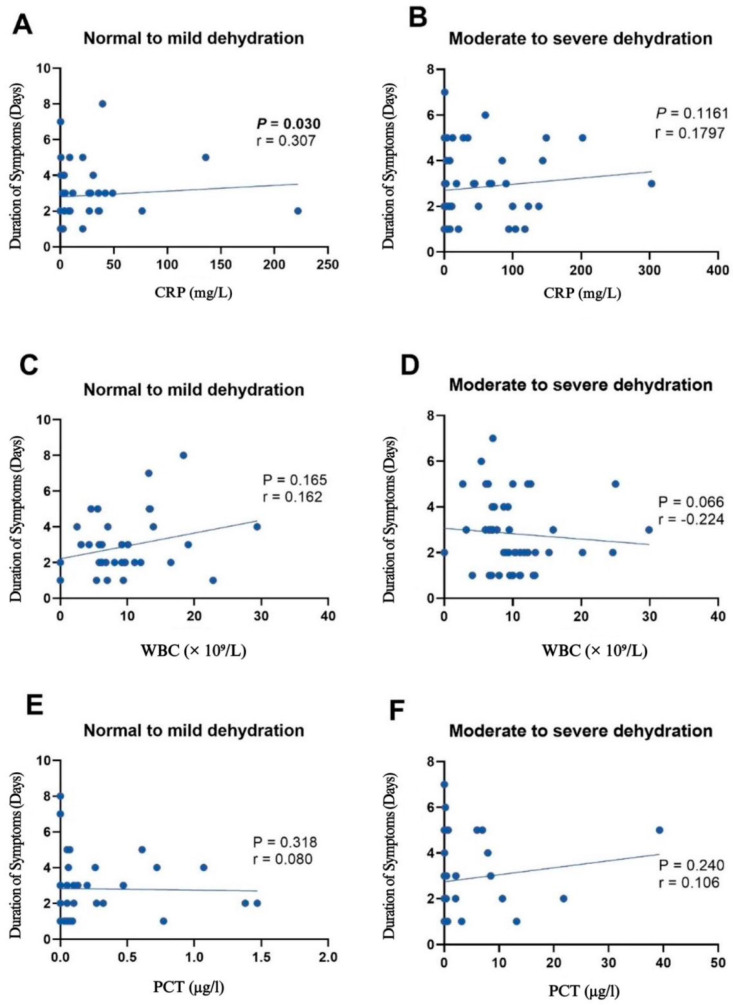
Scatterplots depicting the relationships between various inflammatory markers and symptom duration in patients with normal to mild dehydration (**A**,**C**,**E**) and moderate to severe dehydration (**B**,**D**,**F**). r: Spearman’s coefficient.

**Figure 5:**
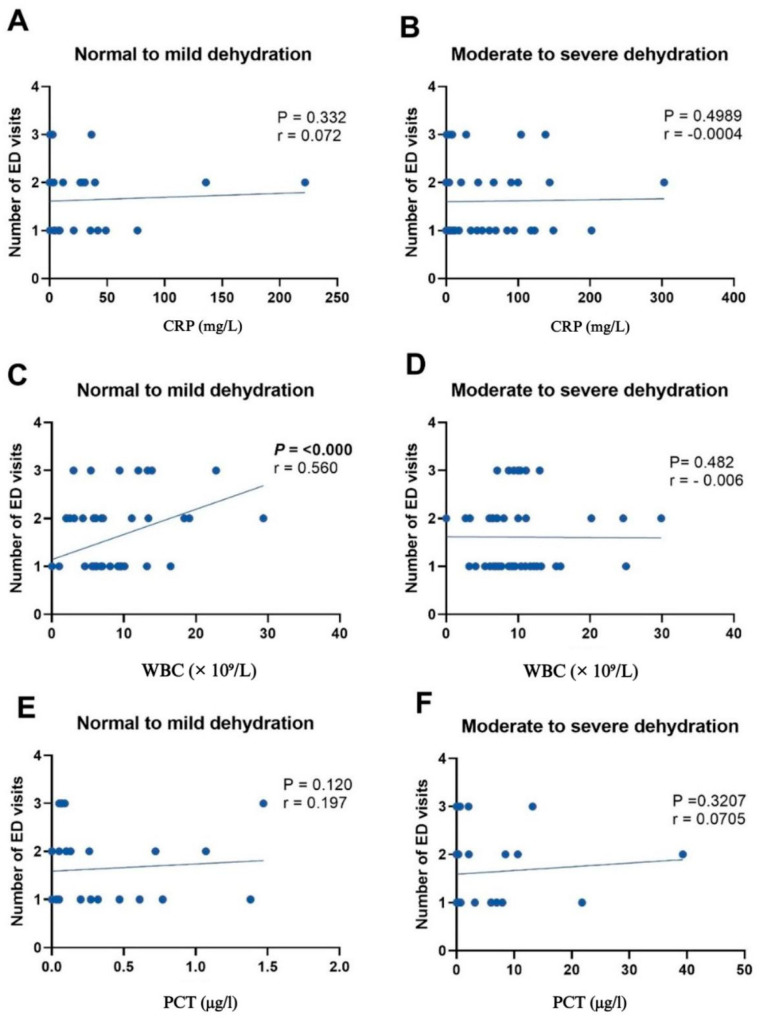
Scatterplots depicting the relationships between the number of emergency department (ED) visits and C-reactive protein (CRP), white blood cell (WBC), and procalcitonin (PCT) levels in patients with normal to mild dehydration (**A**,**C**,**E**) and moderate to severe dehydration (**B**,**D**,**F**). r: Spearman’s coefficient.

Figure 4 and Figure 5 show scatterplots that provide a comprehensive analysis of the relationships between three key inflammatory markers—WBCs, CRP, and PCT—and their association with the duration of symptoms and number of ED visits for patients with normal to mild dehydration, as well as moderate to severe dehydration. The analysis was conducted using Spearman’s rank correlation. Thus, the Spearman’s rank correlation coefficients (ρ) and *p*-values are provided for each marker–symptom duration relationship, allowing for a comprehensive evaluation of the strength and significance of this association across the dehydration severity spectrum.

In contrast to the lower or less significant correlation seen with the WBC count, Spearman’s correlation analysis generally shows a higher and more significant positive association between the inflammatory markers CRP and PCT and symptom duration. Panel A in Figure 4 showcases a statistically significant positive relationship between CRP levels and the duration of symptoms in the normal to mild dehydration group (Spearman’s rho, ρ = 0.307, *p* = 0.030). However, in the moderate to severe dehydration group, this relationship was not statistically significant. WBCs initially had a positive relationship with normal to mild dehydration severity, but in moderate to severe dehydration, an inverse relationship with weak and marginal significance is observed, as shown in panel D (Spearman’s rho, ρ = −0.224, *p* = 0.066).

Compared to the stronger association observed with the WBC counts, the correlation between PCT levels and ED visits is less significant. Panel C in Figure 5 showcases a statistically significant positive relationship between the WBC counts and number of ED visits in the normal to mild dehydration group (Spearman’s rho, ρ = 0.560, *p* = 0.006). However, in the moderate to severe dehydration group, this relationship is not statistically significant. CRP and PCT levels had a negligible relationship with the number of ED visits.

### 3.5. Inflammatory Markers’ Relationships with Extraintestinal Infections and Management Offered

Table 5 and Table 6 provide a multiple logistic regression analysis of the dependent factors (extraintestinal infections and management) and show how numerous independent factors (WBC, Neut, Lymph, CRP, and PCT levels) affect the likelihood of each condition. Both tables show the odds ratios, 95% confidence intervals, and the significance levels for the logistic regression analysis. Each independent value starts with an intercept that represents the baseline or starting point for the relationship between the independent variables (inflammatory markers) and the dependent variable (extraintestinal infections and management). The intercept for extraintestinal infection is statistically significant in all dependent variables. However, when identifying each dependent variable, not every inflammatory marker (WBCs, Neuts, Lymphs, CRP, and PCT) shows a significant relationship with LRTI, URTI, sepsis, and UTI, as indicated by their *p*-values. The intercept for the management that was received is only significant for the use of antibiotics. Furthermore, higher CRP levels are associated with an increased use of antibiotics.

## 4. Discussion

Acute gastroenteritis (AGE) is defined as a change in the consistency of stool to a loose form, and/or an increase in the frequency of defecation. It may or may not be accompanied by fever or vomiting [2]. A considerable link between mortality and morbidity and AGE have been well documented and observed in the literature over the years. Although diarrhea in children is common and mainly self-limited, acute infectious diarrhea is described as the second leading cause of death globally in children below the age of five [7]. Hence, the early detection and management of severe disease or complicated presentation are of great value. Many inflammatory markers have been studied in the context of AGE, mainly as tools to predict its clinical course and determine the need for antimicrobial usage [8]. This study focused on CRP, PCT, and WBC counts as inflammatory markers of AGE and aimed to highlight their relation to AGE’s etiology, the duration of symptoms, ED visits, and the severity of the disease.

When it comes to WBC counts, previous studies have reported that the WBC differentials count is of value in distinguishing between bacterial and non-bacterial AGE early on [9]. In our study, WBC counts were significantly correlated with the duration of symptoms and number of ED visits in the mildly dehydrated group. Thus, WBC counts can possibly aid pediatricians in predicting those likely to return to the ED. On the other hand, CRP and PCT’s relationship with ED visits was negligible.

Our study is consistent with previous studies regarding the value of CRP in identifying patients who are likely to have bacterial AGE [10,11]. It has also been noted that higher levels of CRP correlate with a higher use of antibiotics. As with CRP, the literature on PCT has mostly found it useful in identifying bacterial AGE [12,13]. However, our study does not demonstrate more significant findings regarding PCT and its relationship with this study’s variables. Rui-Mu Zhang’s study compared a group with enterovirus infection to a group with a bacterial co-infection and found no statistically significant difference between the values of inflammatory markers (WBCs, CRP, and PCT) in both groups [14]. It is worth noting that the patients in our study who had a co-infection with AGE showed no significant difference in any inflammatory markers.

Only 13 patients had an identified pathogen. Of that number, nine patients had a bacterial pathogen, mostly *Salmonella*. Due to the limited identification of pathogens in ED visitors, we were not able to fully highlight how different pathogens impacted inflammatory markers. However, a study by Park et al. found that CRP is the only marker that correlates with both fever and bacterial etiology. They have also demonstrated that if combined with fecal lactoferrin, the diagnostic capability of CRP for bacterial infections is higher [1]. Pavia et al. conducted a multicenter, prospective pre- and post-intervention study to evaluate the direct and indirect impact of the introduction of a multiplex molecular diagnostic panel on the management of acute gastroenteritis in children. It is evident from their study that in children who present to the ED with AGE, testing that uses a multiplex molecular diagnostic panel is associated with a 21% reduction in the likelihood of return visits to healthcare providers compared with a clinical diagnosis [15]. Furthermore, their article emphasizes the importance of pathogen identification in guiding management and withholding empirical antibiotics.

Empirical antimicrobials were used in only 29% of the cases. This finding is not unusual, as routine antimicrobial therapy in AGE is not recommended, as per ESPGHAN’s 2014 guidelines. Few pathogens and medical conditions dictate the necessity of antimicrobials for AGE. *Salmonella*—which is the most common bacterial pathogen in our sample—are not treated with antimicrobials, except in high-risk patients. As described in the literature and emphasized in ESPGHAN’s guidelines, the use of antimicrobials in low-risk *Salmonella* cases does not influence symptoms or complications. Furthermore, antimicrobial treatment is recommended in cases of *Shigella*, *Enterotoxigenic E.coli*, and *dysenteric Campylobacter*.

The major limitation of our study is its sample size, which has been an obstacle in pooling further information about the inflammatory markers that were studied, particularly for disposition decisions. The poor documentation of ED presentation has contributed to this limitation, as we had to exclude some patients due to the lack of clinical documentation for their visit. Hence, proper documentation is a necessity for patient care and for future studies. The other limitation was that not all cases were tested for underlying causative pathogens.

## 5. Conclusions

In conclusion, this study highlights that CRP is of great value in helping pediatric emergency physicians identify patients who are likely to have bacterial AGE and require antibiotics. Moreover, the WBC count is a helpful tool in predicting those likely to present to the ED again. Thus, based on these results, we recommend the use of WBCs and CRP in AGE cases presenting to the ED to aid and facilitate proper disposition and management. A larger sample size is required to further investigate the relationship between the organisms cultivated in culture and the severity of the disease.

## Figures and Tables

**Table 1:** Demographic characteristics.

Age in Months:	
Mean ± SD	47.05 ± 45.54
**Medical Background:**	
n (%) Normal	51 (62.07%)
n (%) Abnormal	33 (37.93%)
**Gender:**	
Male: 41 (48.81%)	Female: 43 (51.19%)

**Table 2:** Clinical presentation.

Number of ED Visits	
n (%)	Once: 58 (69.05%) Twice: 19 (22.62%) Three Times: 7 (8.33%)
**Frequency of diarrhea (episodes/day)**	
Mean ± SD	4.64 ± 3.13
**Frequency of vomiting (episodes/day)**	
Mean ± SD	3.70 ± 2.33
**Duration of symptoms (days)**	
Mean ± SD	2.84 ± 1.57
**Fever**	
n (%)	No: 26 (31%) Yes: 58 (69%)
**Bloody diarrhea**	
n (%)	No: 75 (89%) Yes: 9 (11%)
**Tachycardia**	
n (%)	No: 42 (50%) Yes: 42 (50%)
**Hypotension**	
n (%)	No: 81 (96%) Yes: 3 (4%)
**Hypoglycemia**	
n (%)	No: 80 (95%) Yes: 4 (5%)
**Initial dehydration severity**	
n (%)	Normal–mild: 39 (46%) Moderate: 36 (42%) Severe: 10 (12%)

**Table 3:** Summary of inflammatory markers.

Inflammatory Markers:	
**WBC**	
Mean ± SD (Normal range: 5.0–13.0 × 10^9^/L)	9.67 ± 5.92
**Neut**	
Mean ± SD (Normal rove: 2.0–7.5 × 10^9^/L)	5.70 ± 4.65
**Lymph**	
Mean ± SD (Normal range: 1.0–4.0 × 10^9^/L)	3.60 ± 7.61
**CRP**	
Mean ± SD (Normal range: <20 mg/L)	36.33 ± 56.8
**PCT**	
Yean ± SD (Normal range: <0.05 µg/L)	1.98 ± 6.25
**ESR**	
Mean ± SD (Normal range: <20 mm/h)	5.73 ± 17.75

**Table 4:** Summary of viral and bacterial etiology.

Etiology	Cases	Total Cases	Prevalence (%)
**Viral Etiologies**			
*Rotavirus*	1	84	1.19
*Norovirus*	1	84	1.19
**Bacterial Etiologies**			
*Salmonella*	6	84	7.14
*Shigella*	1	84	1.19
*E. coli*	1	84	1.19
*Blastocystis hominis*	1	84	1.19
**Mixed infection**			
*Enteroaggregative E. coli* + *Astrovirus*	1	84	1.19
*C. difficile Norovirus Sapovirus*	1	84	1.19

**Table 5:** Multiple logistic regression analysis of dependent variables (extraintestinal infections) and independent factors (inflammatory markers).

Dependent Variable	Independent Variable	Odds Ratio	95% CI	* *p* Value
**LRTI**	Intercept	0.010	0.000 to 0.113	**0.001**
	WBC	4.725	1.126 to 36.11	0.065
	Neut	0.190	0.018 to 0.988	0.084
	Lymph	0.171	0.010 to 1.037	0.125
	CRP	1.006	0.988 to 1.022	0.408
	PCT	0.999	0.724 to 1.145	0.993
**URTI**	Intercept	0.195	0.062 to 0.556	**0.003**
	WBC	1.454	1.090 to 3.756	0.417
	Neut	0.642	0.220 to 0.901	0.391
	Lymph	0.845	0.269 to 1.059	0.768
	CRP	0.997	0.988 to 1.00	0.563
	PCT	1.085	1.003 to 1.210	**0.067**
**Sepsis**	Intercept	0.025	0.003 to 0.144	**0.000**
	WBC	1.164	0.837 to 3.892	0.662
	Neut	0.917	0.232 to 1.391	0.827
	Lymph	0.938	0.190 to 1.071	0.879
	CRP	1.002	0.984 to 1.014	0.799
	PCT	1.047	0.916 to 1.145	0.354
**UTI**	Intercept	0.052	0.003 to 0.423	**0.015**
	WBC	2.003	0.431 to 17.10	0.483
	Neut	0.514	0.045 to 3.022	0.554
	Lymph	0.248	0.010 to 1.042	0.327
	CRP	1.009	0.994 to 1.024	0.199
	PCT	0.912	0.514 to 1.079	0.582

* Multiple Logistic Regression Analysis.

**Table 6:** Multiple logistic regression analysis of dependent variables (management lines) and independent factors (inflammatory markers).

Dependent Variable	Independent Variable	Odds Ratio	95% CI	* *p* Value
**Oral challenge**	Intercept	1.435	0.565 to 3.699	0.447
	WBC	0.604	0.252 to 1.032	0.231
	Neut	1.788	0.967 to 4.736	0.217
	Lymph	1.735	0.982 to 5.060	0.289
	CRP	0.996	0.986 to 1.005	0.354
	PCT	1.107	0.981 to 1.386	0.220
**Regular IV Fluid**	Intercept	2.521	0.705 to 9.549	0.160
	WBC	0.657	0.209 to 1.115	0.469
	Neut	1.940	1.022 to 7.609	0.323
	Lymph	1.521	0.935 to 6.245	0.552
	CRP	1.001	0.981 to 1.016	0.847
	PCT	1.012	0.911 to 1.243	0.841
**NS Bolus**	Intercept	0.925	0.273 to 2.948	0.897
	WBC	0.575	0.190 to 1.059	0.296
	Neut	2.107	1.042 to 7.420	0.212
	Lymph	1.743	0.932 to 6.495	0.386
	CRP	1.004	0.988 to 1.025	0.648
	PCT	11.270	1.160 to 797.2	0.168
**D10W Bolus**	Intercept	0.133	0.012 to 0.880	0.060
	WBC	0.885	0.541 to 3.636	0.612
	Neut	1.141	0.229 to 2.020	0.640
	Lymph	1.020	0.163 to 1.332	0.918
	CRP	1.003	0.973 to 1.022	0.797
	PCT	1.045	0.334 to 1.016	0.346
**Antibiotics**	Intercept	0.154	0.047 to 0.448	**0.001**
	WBC	0.916	0.493 to 1.118	0.470
	Neut	1.185	0.919 to 2.368	0.258
	Lymph	1.055	0.968 to 2.283	0.594
	CRP	1.012	1.003 to 1.024	**0.022**
	PCT	1.091	1.001 to 1.254	0.094

* Multiple Logistic Regression Analysis.

## Data Availability

Data is available upon request from the corresponding author due to legal reasons.

## Abbreviations

The following abbreviations are used in this manuscript:

AGE	Acute gastroenteritis
WBCs	White blood cells
Neuts	Neutrophils
Lymphs	Lymphocytes
CRP	C-reactive protein
PCT	Procalcitonin
ESR	Erythrocyte sedimentation rate
URTI	Upper respiratory tract infection
LRTI	Lower respiratory tract infection
UTI	Urinary tract infection
ED	Emergency medicine
ESPGHAN	European Society for Pediatric Gastroenterology, Hepatology, and Nutrition
AAP	American Academy of Pediatrics
